# The Secrets of the Panel

**Published:** 1921-01-29

**Authors:** 


### The Secrets of the Panel.

The agitation against the system of medical record cards for panel patients is continuing, though on a lower note of protest. *The Times*, which for some reason or other has worked itself up into a great state of indignation in this matter, and has assumed the role of champion of the "anti-record" party, keeps returning to the charge, and has now even reached the point of giving the weight of the

**THE PUBLIC WELL-BEING**—(*continued*).

stationery involved in the work in certain areas. It also bemoans the fate of panel doctors who will now have to keep a filing cabinet, and even, it seems, an alphabetical index. And what will they do, asks *The Times*, when there is an epidemic of influenza? This wail concludes with the usual statement, quite unjustified by any known facts, that the records are not likely to serve their intended purpose, and with the suggestion, which is dangerously akin to an invitation, that "sensitive panel patients" will in future withhold essential features of their condition or prefer to be treated as private patients.

On these lines the controversy threatens to enter the region of the ridiculous, and one prefers, by contrast, the sound and sensible position taken up by the Medico-Political Union, of Gray's Inn Square, which represents 3,500 doctors, and has sent the Minister of Health a letter dealing with the new system. In the words of Dr. Welply, the Secretary of the organisation, the communication is not a protest, but contains constructive suggestions with regard to the difficulties as to secrecy and clerical work. We have said before that Dr. Addison would be well advised to seek the assistance of panel doctors as a body on the details of the system, for when once he has secured their co-operation the rest will be easy, because patients as a whole will accept the assurance of their own doctors that no secrets of their medical history will be disclosed. Dr. Welply takes up a perfectly reasonable attitude. "Doctors recognise," he says, "that some records must be kept, and believe that they can be modified in such a way that the doctor himself would be relieved of a great deal of the drudgery which in their present form the records impose. If Dr. Addison had consulted at the outset the men who are doing the work, the difficulty would never have arisen. With reasonableness on both sides the obstacles can still be overcome without the efficacy of the records being impaired."

Dr. Welply laid stress on the danger of medical records being used in evidence in the divorce courts and in litigation of a kindred character. In our view, that is the one solid objection which is not easily to be met. The difficulty was strikingly exemplified a few days ago in a case in which a medical officer of the Bethnal Green Health Committee was ordered under a subpœna of the Crown to produce in Court intimate information with regard to the birth and after-care of a certain child. The required information was in his possession as a public official connected with the maternity side of public health. Both the medical officer and his Committee were quick to see the disagreeable possibilities arising from an order of the Court of this description, and they appealed to the Ministry of Health for advice as to their legal position in the matter. The Ministry replied, with apparent reluctance, that it was doubtful whether such documents in the possession of a local authority could be regarded as privileged. That being so, it is quite evident that not only the panel record cards, but the information available at public clinics are all open to public exposure in Courts of law. It is not difficult to see how unsatisfactory it would be if this impression became generally diffused. The work of both clinics and panel doctors might be gravely hampered, because the tendency would undoubtedly be on the part of a large section of the public to keep back important information, with ultimately unfortunate results to the public health. The clear solution is that an authoritative pronouncement should be made constituting all such documents as "privileged." We have little doubt that sooner or later this will have to be done.

